# Emotional and Social Mind Training: A Randomised Controlled Trial of a New Group-Based Treatment for Bulimia Nervosa

**DOI:** 10.1371/journal.pone.0046047

**Published:** 2012-10-31

**Authors:** Anna Lavender, Helen Startup, Ulrike Naumann, Nelum Samarawickrema, Hannah DeJong, Martha Kenyon, Frederique van den Eynde, Ulrike Schmidt

**Affiliations:** 1 Eating Disorders Service, South London and Maudsley NHS Foundation Trust, Maudsley Hospital, London, United Kingdom; 2 Department of Biostatistics and Computing, Institute of Psychiatry, King's College London, London, United Kingdom; 3 Section of Eating Disorders, Department of Psychological Medicine, Institute of Psychiatry, King's College London, London, United Kingdom; University of Pennsylvania, United States of America

## Abstract

**Objective:**

There is a need to improve treatment for individuals with bulimic disorders. It was hypothesised that a focus in treatment on broader emotional and social/interpersonal issues underlying eating disorders would increase treatment efficacy. This study tested a novel treatment based on the above hypothesis, an Emotional and Social Mind Training Group (ESM), against a Cognitive Behavioural Therapy Group (CBT) treatment.

**Method:**

74 participants were randomised to either ESM or CBT Group treatment programmes. All participants were offered 13 group and 4 individual sessions. The primary outcome measure was the Eating Disorder Examination (EDE) Global score. Assessments were carried out at baseline, end of treatment (four months) and follow-up (six months).

**Results:**

There were no differences in outcome between the two treatments. No moderators of treatment outcome were identified. Adherence rates were higher for participants in the ESM group.

**Discussion:**

This suggests that ESM may be a viable alternative to CBT for some individuals. Further research will be required to identify and preferentially allocate suitable individuals accordingly.

**Trial Registration:**

ISRCTN61115988

## Introduction

Bulimia nervosa (BN) is a common and disabling disorder, particularly in young women [1], [2] with a high burden on the individual, their families and society [3], [4], [5]. Treatment for BN has undergone significant development over the last decade. Cognitive Behaviour Therapy (CBT) for BN has proved an effective treatment for many sufferers, and a specific form of CBT (CBT-BN) [6], [7], is the first-line psychological treatment recommended by the National Institute for Clinical Excellence (NICE). Individual and group formats of this treatment have been tested, with comparable efficacy [8], [9].

However, although promising, only 30 to 40% of people are symptom free at the end of treatment with CBT [10]. A recognition of the need to improve treatment outcomes has led to the development of a form of CBT designed to address a broader range of putative maintaining factors [11]. However a large RCT (n = 154 patients) comparing the more focused (CBT-Ef) version of CBT-BN with the broader (CBT-Eb) version failed to show any difference in outcome, either at the end of treatment or at five-year follow up, with half the sample retaining a level of eating disorder pathology one standard deviation above the community mean [12]. Thus, the need to develop more effective treatments for this condition remains.

### Exploring New Targets for Treatment of Bulimia

One possibility is that CBT has over-focused on targeting the overt symptoms of BN (bingeing and compensatory behaviours) at the expense of the broader intra- and interpersonal attitudes, affects and behaviours typical of the disorder. The potential utility of focussing on interpersonal difficulties in BN has been recognised in the development of Interpersonal Psychotherapy (IPT) for BN [13], for which outcomes are comparable to those seen in CBT (e.g. [14], [15]), albeit at a slower pace. IPT focuses on one of four possible domains of interpersonal difficulties for an individual. In a recent systematic review which explored factors predicting poor outcome in eating disorders, two key factors emerged for BN: poor social/interpersonal functioning and negative self-evaluation [16]. Thus while IPT does address interpersonal functioning, its focus on one particular domain for an individual means that the broader social and emotional context of these difficulties and how they relate to an individual's sense of self, are not addressed.

Dialectical behaviour therapy, which does emphasise interpersonal difficulties, emotional dysregulation and distress tolerance, has recently been adapted for individuals with BN and borderline personality disorder. In a small study of this treatment, Chen et al [17], found large effects in terms of reductions in bulimic symptoms. However this treatment involves a much greater therapeutic input than is possible within most outpatient contexts, involving six months of individual therapy, six months of group therapy, and 24 hours of telephone coaching…

#### Social/interpersonal functioning in Bulimia

There is substantial evidence to suggest that many women with BN experience difficulties in the social domain during childhood [18], [19]. Severe life events and chronic difficulties in the social/interpersonal domain often trigger the onset of BN in the majority of cases [20], [21].

Women with established BN often compare themselves unfavourably to other women and perceive themselves to have a lower social rank than others [22], [23]. Moreover, they are more likely to have a more limited social network with fewer supportive relationships [24]. Pre-existing social difficulties may be worsened by the effects of bulimic symptoms, such as semi-starvation, bingeing or compensatory behaviours [25].

In the treatment literature, Leung *et al.* [26] found that amongst women treated for BN with group based CBT, it was those who held non-eating disorder-related pathological core beliefs at the outset of treatment who ended up making fewer treatment gains. These included difficulties in the realm of interpersonal functioning and low self-esteem. Thus, there is growing evidence that social functioning and social cognition are impaired in BN and may provide fruitful targets for treatment.

#### Negative self-evaluation, shame and social threat

A consistent finding within the literature concerns the emphasis on affect as a key maintaining factor for BN [27]. Wonderlich et al, 2008 [28] found that negative mood states mediated the relationship between bulimic symptoms and negative self-directed coping styles. Waller *et al.* [27] suggest that while anorexia nervosa is characterised by primary avoidance (preventing the experience of distressing cognitions and emotions), BN is characterised by secondary avoidance (removing or blocking the experience of distressing emotions and cognitions). One way of understanding the link between emotion and negative self-evaluation in BN may be through the concept of shame-based self-criticism [29]. Eating disordered individuals have been found to have high levels of both internal shame (self devaluation and self-criticism) and external shame (negative thoughts and feelings about how one exists in the mind of others) [30], [31].

#### Social Cognition and Bulimia Nervosa

Social cognition (SC) refers to the mental processes that underlie social behaviour and interpersonal interaction. It involves the ability to perceive and understand the feelings, intentions and beliefs of others, and to use this information to guide social behaviour. It also encompasses emotional processing, which includes identifying, utilising, understanding and managing emotions, both in relation to oneself and others. A recent systematic review identified only a handful of studies on this topic in relation to bulimia nervosa [32]. The review found that people with BN are not significantly impaired in performing basic emotion recognition or theory of mind tasks. They were however impaired at inferring the emotions of self and others in interpersonal scenarios [33], and they showed greater accuracy than healthy people at identifying negative emotions [34]. They also had enhanced attention towards angry faces [35]. Considering the aspect of social cognition that relates to managing emotions, Fischer and colleagues (e.g. [36], [37]) have identified that individuals with BN tend to respond with rash and ‘urgent’ behaviour when distressed. Taken together these findings suggest an elevated propensity to attend to negative or threatening socio-emotional information.

### A New Emotional and Social Mind Group Training Programme

Taken together the above evidence suggests that negative self-evaluation, poor interpersonal skills, difficulties in understanding the minds of others (the ‘social mind’), a propensity to attend to negative or threatening socio-emotional information and difficulties managing and tolerating emotions, particularly those linked with shame, may be key factors in the maintenance of bulimic symptoms. Based on these ideas we have developed a new emotional and social mind group training programme for individuals with BN and related disorders. Because we believe that shame and interpersonal difficulties are central to the pathology of these individuals, a group format may provide powerful opportunities to normalise experiences, learn from others and explore the ‘minds’ of others within a safe environment. We hypothesise that a treatment targeting these factors will lead to greater positive changes in self-evaluation, interpersonal functioning, and mood, and a greater reduction in bulimic symptoms than a group-based ‘standard’ CBT programme.

## Methods

### Design

This was a two-arm randomised controlled trial designed to evaluate the efficacy of Emotional and Social Mind Training (ESM) compared to group CBT for adults with BN consecutively referred to the South London and Maudsley (SLaM) NHS Foundation Trust Eating Disorders Outpatient Service. The protocol for ths trial and supporting CONSORT checklist are available as supporting information; see Checklist S1 and Protocol S1.

### Participants

*Inclusion criteria*: Patients consecutively referred to the SLaM Eating Disorders Outpatient Service by their general practitioner were offered participation if they were (a) aged 18–60, (b) fulfilled the Diagnostic and Statistical Manual of Mental Disorders (DSM-IV) criteria for BN or EDNOS as assessed by a clinician specialising in the treatment of EDs. EDNOS differed from BN in that it required at least 6 binge eating episodes in the last 28 days, a minimum binge frequency that lies between the current DSM-IV criteria for BN (twice per week) and those proposed for DSM-5 (once per week) [38].

#### Exclusion Criteria

Patients were excluded if they had insufficient knowledge of English or literacy levels to allow understanding of the intervention materials, active suicidality, current substance dependence, diabetes or pregnancy.

#### Recruitment

Recruitment took place between March 2009 and November 2010. Informed written consent was gained from patients at baseline assessment. The study was approved by the Joint South London and Maudsley and Institute of Psychiatry Research Ethics Committee, Study reference number 08/H0807/83.

### Interventions

*Commonalities between treatments:* In both arms patients received a 17-session treatment (4 individual sessions, 12 group sessions, 1 follow-up session). The follow-up session was a ‘booster’ for the group. Group sessions took place on a weekly basis. Individual sessions were 60 and group sessions 90 minutes in length. Group sessions included eight patients and were facilitated by two therapists. The ESM and CBT group timescales and group makeup were identical.

### Emotional and social mind training (ESM)

We developed a manual for ESM that specified the content of each of the twelve sessions, divided into three stages, of the programme, which was designed to address the hypothesised maintaining factors outlined above.

In the first stage (sessions 1–5), identification and understanding of inter- and intra-personal emotions, the social context of emotions and understanding others' emotions were key themes, as were identifying and understanding difficulties with self-esteem and the role of BN as a coping strategy. The rationale and model underpinning ESM was shared and explained. Patients were encouraged to engage in weekly monitoring of their BN symptoms and to begin to explore the link between their emotions, self-esteem and their BN.

In stage two (sessions 6–10) developing alternative, ‘non BN’ ways of coping, was the core theme. This included practice of developing self compassion to manage self-criticism and shame, exploring ways to managing intense and overwhelming emotions, education about and practice of alternative coping strategies, and exploration of ways to nourish oneself beyond BN.

Stage 3 (sessions 11–12) was concerned with consolidation of therapeutic gains and relapse prevention strategies. The follow-up group session, which took place approximately two months after the final weekly group, focused on relapse prevention and maintenance. The individual sessions were used to develop an individual case formulation, ‘troubleshoot’, and individualise learning from the groups.

#### CBT

The intervention was based on the group CBT treatment for BN developed by Chen *et al.* [8], adapted from Fairburn *et al.* [7]. We chose this programme as the comparison treatment as we wished to match the group format of our ESM intervention whilst comparing it to CBT. We adapted Chen et al's [8] treatment manual for the purposes of our study.

### Therapists

Groups were run by 16 experienced therapists who had attended training workshops in both group ESM and CBT prior to the study.

### Treatment fidelity

Therapists attended weekly supervision with experienced senior supervisors to ensure both treatments were delivered competently and uniformly. All therapeutic sessions were taped and a random selection of these listened to by the research team to check for adherence to therapeutic model and quality of intervention.

### Assessments

Patients in both groups met with a research worker to complete research assessments at baseline, four months (end of weekly treatment) and six months (follow-up).

#### Eating Disorder

Severity of core bulimic symptoms was assessed using the Eating Disorder Examination (EDE; [39]), a widely used semi-structured clinical interview, considered to be the gold standard to assessing ED symptomatology. The primary outcome was EDE Global score. Secondary outcomes included the four EDE subscales (restraint, eating concern, weight concern and shape concern), and objective binge episodes and episodes of self-induced vomiting in the previous four weeks.

#### Other psychopathology and demographics

Secondary outcomes of comorbid depression and anxiety were assessed using the Depression, Anxiety and Stress Scale (DASS-21; [40]. Quality of life was assessed using the Clinical Impairment Assessment (CIA; [41]). Measures of other psychopathology linked to the ESM model maintaining factors were also included. Levels of self-criticism were assessed using the Levels of Self-Criticism Scale [42], which consists of two subscales, internalized self criticism (LOSCISC) and comparative self criticism (LOSCCSC). Submissiveness was assessed using the Submissive Behaviour Scale (SBS) [43]. Negative beliefs about expressing emotions were measured using the Beliefs About Emotions Scale (BES) [44]. Ability to tolerate distress was assessed using the Distress Tolerance Scale (DTS) [45], which consists of three subscales: Anticipate and Distract (DTS1), Avoidance of Affect (DTS2) and Accept and Manage (DTS3).

### Sample size

Because ESM was a novel intervention, power was calculated on the basis of Chen *et al.* [8]. A sample size of 30 in each arm would have 80% power to detect a difference in means of −0.850 on the EDE Global score, assuming that the common standard deviation is 1.15 using a two group t-test with a 0.05 two-sided significance level. Assuming a drop-out rate of 20%, the required sample size was 38 patients per group.

### Randomisation, Blinding and Protection against Bias

Once the assessing clinician ascertained a patient was suitable for the trial and consent had been obtained, she/he was introduced to the research assessor who completed the research assessment. The patient was then be randomised by a computerised system. Randomisation was stratified for diagnosis (BN or EDNOS) and patients were assigned to one of the two trial arms. Patients were told about the outcome of randomisation by the assessing clinician. Outcome assessments were conducted by research assessors blind to the treatment condition.

### Statistical analysis

#### Mixed Model

Our data consisted of three time-points, baseline, post-treatment (4 months) and follow-up (6 months). The aim was to investigate differences between the intervention groups at each post treatment time point for each of the outcomes.

The outcome variables at the 4 and 6 month post-randomisation time points constituted the dependent variables in our model. Pre-randomisation (baseline) values of the outcomes were used as covariates in the respective models to reduce bias of the estimates.

Mixed models were used to simultaneously model multiple post treatment outcomes of a scale or subscale. The correlation between the individual observations at post-treatment and follow-up was taken into account by including random Intercepts in the models. The fixed part of the model contained the covariates treatment arm (0 = CBT, 1 = ESM), time (0 = 4 months, 1 = 6 months) and an interaction of group and time (coded 1 = 6 months & ESM, 0 = else) to represent the additional increase of outcome from 4 to 6 months in the ESM group. For some of the outcomes, i.e. EDE restraint the time-group-interaction was not included in the model, since it was very small and not significant. We also controlled for the stratification variable Diagnosis (BN or EDNOS) by including it in the model as a covariate.

The questionnaire outcomes were assumed to arise from normal distributions.

Distributions were assessed using diagnostic plots. We transformed the outcomes ‘ED behaviours objective binges’ and ‘ED behaviours vomiting’ using a log-transformation, and transformed the EDE shape score and attendance (number of group sessions attended) using a square transformation, so that the assumption of the normal distribution was correct.

The binary outcome ‘EDE in normal range’ was defined as EDE total smaller/larger than 1.7 (normal range = 1/0). Abstinence was defined as the absence of any objective binges or episodes of vomiting within the previous month. For the binary outcomes, abstaining and ‘EDE in normal range’, we used logistic mixed regression models respectively.

The longitudinal analyses were done using the Intention To Treat (ITT) principle Our analysis included some additional outcomes, the individual session attendance and (squared) group attendance. The purpose of the analyses of individual and group session attendance was to measure whether adherence to treatment was different between the two groups.

The individual session attendance was measured as the number of individual psychotherapy sessions that the patient attended, and the group attendance was measured by the number of group sessions that the patient attended. The group attendance was transformed using a square-transformation in order to ensure that the assumption of a normally distributed outcome was met.

We used an ordered logistic regression to model individual session attendance and a linear regression to model the (squared) group attendance.

#### Multiple Imputation

We had both missing data for some of the outcomes at post-treatment and follow-up (research drop-out), as well as drop out from treatment. If missing outcome variables are missing at random (MAR), i.e. the missing data mechanism is ignorable, a linear mixed model will still provide unbiased estimates. However, we found that adherence to the treatment (measured by the attendance of group and individual sessions) predicted dropout from the trial. We therefore used Multiple Imputation with 5 imputations applying a set seed using the ice package in Stata version 11.2, to ensure that our analysis would provide unbiased estimates. The imputation model included all variables of the primary and secondary analysis, as well as all variables used in the moderator analysis. These were in detail, the outcome variables of the main analysis at baseline, post-treatment and follow-up, as well as individual and group session attendance, the treatment group, diagnosis, the moderator variables at baseline, demographic variables and BMI at baseline.

The estimates from the regression were drawn from the average of analysis of each of the imputed datasets (n = 5) and are combined using Rubin's Rule [46] and the ‘mim’ command in Stata.

## Results

### Patient flow

**Figure 1:**
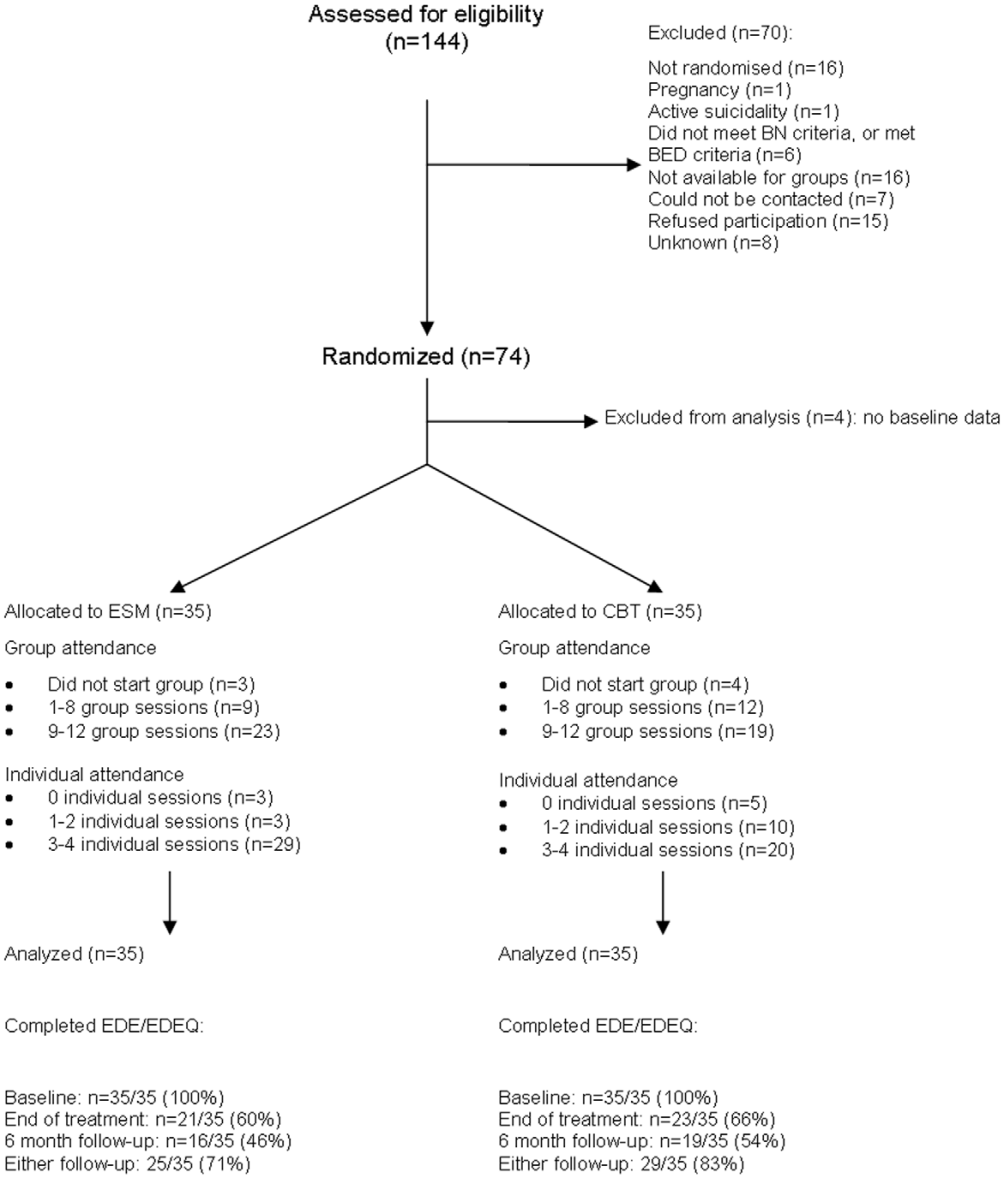
Consort diagram.

Figure 1 shows the participant flow through the study. 74 patients were randomized, 37 to ESM and 37 to CBT. Two patients from each arm were excluded from analysis due to lack of baseline data. This was due to these participants accidentally being randomised prematurely when the clinician took consent before the baseline research assessment.

### Patient Characteristics at baseline

Patients allocated to ESM and CBT did not differ significantly in terms of any recorded baseline demographics or clinical characteristics (see Table 1).

**Table 1:** Baseline Characteristics.

	ESM		CBT	
	N	Mean (SD) or N (%)	N	Mean (SD) or N (%)
**Demographic details**				
Age	35	27.7 (7.6)	35	27.7 (7.3)
Gender (male∶female)	35	1∶34	35	4∶30
Ethnicity	35		35	
White British		28 (80%)		26 (74%)
White Other		3 (9%)		4 (11%)
Black British		0 (0%)		1 (3%)
Black Other		0 (0%)		2 (6%)
Asian British		0 (0%)		0 (0%)
Asian Other		2 (6%)		1 (3%)
Other		2 (6%)		0 (0%)
NART IQ estimation	32	107.1 (7.5)	35	107.9 (7.1)
**Clinical details**				
Diagnosis	35		35	
BN		19 (54%)		22 (63%)
EDNOS		16 (46%)		13 (37%)
History of AN	34		34	
Yes		3 (9%)		4 (12%)
No		31 (91%)		30 (8%)
Age of onset (years)	33	18.5 (6.2)	29	16.2 (4.0)
Duration of Illness (years)	33	9.3 (7.6)	29	11.4 (7.6)
EDE Global	35	3.8 (1.0)	35	3.9 (1.0)
EDE restraint	35	3.1 (1.5)	35	3.4 (1.3)
EDE eat	35	3.3 (1.6)	35	3.5 (1.3)
EDE weight	35	4.4 (1.4)	35	4.4 (1.3)
EDE shape	35	4.6 (1.2)	35	4.8 (1.0)
Objective binge episodes (past month)	35	18.5 (23.0)	35	19.8 (24.8)
Vomit episodes (past month)	35	24.5 (35.6)	35	21.6 (32.1)
Laxative episodes (past month)	35	8.0 (16.1)	35	4.4 (11.9)
BMI	31	24.4 (5.7)	34	25.3 (7.7)
Medication for depression	35		34	
Yes		12 (34%)		13 (37%)
No		23 (66%)		21 (60%)
DASS Total	33	31.1 (10.9)	31	32.5 (10.9)
DASS Depression	33	10.8 (5.7)	31	12.1 (5.4)
DASS Anxiety	33	8.2 (3.8)	31	8.2 (4.4)
CIA	33	31.5 (9.6)	34	33.7 (7.1)

### Treatment Outcomes

Tables 2 and 3 show differences between CBT and ESM at post-treatment and follow-up respectively.

**Table 2:** Difference between CBT (TAU) and ESM at post treatment.

Outcomes linear mixed model	Estimate	Lower CI	Upper CI	p-value
	Difference between two groups			
**Primary outcomes**				
EDE global n = 44	0.101	−0.391	0.593	0.688
**Secondary outcomes**				
EDE restraint n = 44	0.312	−0.222	0.847	0.251
EDE eating n = 44	0.026	−0.676	0.728	0.942
EDE weight n = 44	−0.170	−0.751	0.410	0.563
Square(EDE shape) n = 44	0.894	−4.029	5.818	0.721
Log(ED behaviours binges)n = 42	−0.161	−0.519	0.197	0.377
Log(ED behaviours vomit)n = 42	0.102	−0.343	0.547	0.652
DASS total n = 44	0.595	−4.057	5.248	0.796
DASS stress n = 44	−0.403	−2.211	1.405	0.660
DASS depression n = 44	0.087	−2.30	2.473	0.940
DASS anxiety n = 44	0.809	−1.129	2.747	0.400
CIA total n = 41	−1.024	−4.844	2.795	0.599

Outcomes logistic mixed model	Estimate	Lower CI	Upper CI	p-value
	Odds Ratio of group difference			
Abstained – binary n = 42	2.107	0.197	22.579	0.517
Normal range – binary n = 44	0.766	0.109	5.374	0.772

**Table 3:** Difference between CBT (TAU) and ESM at follow up.

Outcomes	Estimate	Lower CI	Upper CI	p-value
	Difference between two groups			
**Primary outcomes**				
EDE global n = 34	−0.099	−0.612	0.414	0.704
**Secondary outcomes**				
EDE restraint (no interaction included)n = 34	0.312	−0.222	0.847	0.251
EDE eating n = 34	−0.310	−1.008	0.388	0.380
EDE weight n = 34	−0.170	−0.751	0.410	0.563
Square(EDE shape) n = 34	0.894	−4.029	5.818	0.721
Log(ED behaviours binges) n = 32	−0.161	−0.519	0.197	0.377
Log(ED behaviours vomit) n = 34	−0.080	−0.540	0.381	0.732
DASS total n = 32	−2.576	−7.104	1.952	0.257
DASS stress n = 32	−1.009	−3.344	1.326	0.377
DASS depression n = 32	−0.560	−2.415	1.294	0.552
DASS anxiety n = 32	−1.196	−2.800	0.409	0.143
CIA total n = 31	−2.290	−6.199	1.619	0.250

Outcomes logistic mixed model	Estimate	Lower CI	Upper CI	p-value
	Odds Ratio of group difference			
Abstained – binary n = 33	3.458	0.363	32.959	0.267
Normal range – binary n = 34	1.676	0.138	20.321	0.645

It will be noted that for some outcomes, estimates for differences in treatment effect for the two groups are the same at post-treatment and follow-up. This is because the time-group interaction was not included in the analysis in instances where it was small and insignificant. We found no significant difference of the treatment effect on the primary and secondary outcomes between the two treatment groups.

Patients in both treatment groups significantly improved over time on primary and secondary outcome variables, which did not differ significantly between treatments. These gains were maintained at follow-up (data not shown, but can upon request be obtained from the authors).

We found a significant difference in attendance of individual sessions between treatment groups. The odds of patients who attended the ESM group to attend an additional session are 3.5 times as high (CI = [1.284, 9.769], p = 0.015) as the odds of the patients who attended the CBT group.

We found no significant difference in the (squared) group attendance between both treatment groups (16.213, CI = [−4.061, 36.487], p = 0.115). However, if we controlled for the baseline covariates SBS, CIA, EDE global, laxatives, then the (squared) group attendance was significantly different between both groups (24.077, CI = [1.812, 46.341], p = 0.035), so that ESM group participants were likely to attend more group sessions than CBT group participants.

### Exploratory Moderator Analysis

We conducted additional analyses to find out whether the treatment effect on the primary outcome EDE Global at post treatment was moderated by any of the following potential moderators: diagnosis, baseline beliefs about emotions (BES), baseline Distress tolerance scale - anticipate distract (DTS1), baseline Distress tolerance scale - avoidance of affect (DTS2), baseline Distress tolerance scale – accept manage emotions (DTS3), baseline submissive behaviour scale (SBS), baseline internalized self criticism (LOSCISC) or baseline comparative self criticism (LOSCCSC).

We did not consider observations at follow-up for this analysis but only looked at baseline and post-treatment outcomes. With the data from the Multiple Imputations, we used linear regression models for the outcome EDE global at post-treatment. We adjusted for the covariates EDE global at baseline, the type of treatment, the diagnosis, the potential moderator and an interaction between the potential moderator and treatment group. We consider that the moderator modifies the effect of the treatment, if there is a significant interaction between potential moderator and treatment group. We did not find any significant moderator effect. Full details can on request be obtained from the authors.

### Sensitivity Analysis

Our main analysis was an ITT analysis to determine the difference in efficacy of ESM compared with CBT. In order to take treatment non-adherence into account, we conducted a sensitivity analysis on a per-protocol basis, including only those patients who received a sufficient level of treatment (those who completed 8 or more group sessions). We found no significant differences between the two treatments. Full details can on request be obtained from the authors.

## Discussion

This study investigated the efficacy of Emotional and Social Mind Training (ESM) a novel, group-based treatment for bulimia nervosa, compared to group-based CBT for bulimia. We hypothesised that ESM, a non-symptom-based treatment that targets the broader emotional and social deficits often experienced by sufferers of bulimic disorders, would be superior to CBT in terms of treatment outcomes. This hypothesis was not supported. Although both treatments performed well and patients in each group improved significantly, no differences in primary or secondary outcomes were found between the two treatments at the end of treatment or at follow-up. The hypotheses that social and interpersonal functioning would improve significantly more in the ESM group were also not supported. Additionally, none of these variables emerged as moderators of outcome in our analyses.

However, it is of interest that ESM performed as well as CBT, the currently recommended first line treatment modality for the treatment of BN. Abstinence and recovery rates did not differ between groups, and were comparable to those obtained in other studies of group and individual treatment for BN (e.g [8], [9], [47].). This gives grounds for optimism that ESM may be a viable alternative to CBT for the treatment of some individuals with BN.

Group treatment for bulimia is an underdeveloped area within the treatment research field. One key question to be answered is whether group treatment is as effective as individual treatment for BN. In the 1980s and early 1990s, numerous group therapies for bulimia were developed and trialled. A meta-analysis of group treatments for bulimia by Fettes and Peters [48] reported a moderate effect size (0.75), significantly smaller than that for individual therapy. Of note, this meta-analysis included treatments from a number of different psychotherapeutic modalities.

However, Chen *et al.* [8] found no difference between individual and group CBT, although a higher proportion of individual CBT patients were abstinent from bulimic behaviours at the end of treatment, a finding which disappeared at follow-up. Similarly, Katzman *et al.* [9] found no difference in treatment efficacy between individual CBT and group CBT prefaced by four individual sessions. It may be that CBT for bulimia is particularly adaptable for groups, and that ESM, whose theoretical framework and structure is based on fairly recent developments within the broad cognitive behavioural field of research in bulimia, retains this adaptability.

Other considerations of the commonalities and differences between ESM and CBT suggest further areas for investigation. ESM, like CBT, is a structured, collaborative therapy based on a model of bulimia shared with the patient. Our clinical sense is that this structure and collaboration, particularly within a group setting, are an essential part of patients feeling safe and contained within therapy.

However, unlike CBT, ESM is less symptom- and more emotion and interpersonally-focused. It targets the broader pathology associated with and hypothesised to underlie BN, including poor social functioning, low self-esteem and poor emotional regulation. It focuses more on group processes, particularly interactions between group members. It also uses some experiential techniques, for example therapeutic writing.

As discussed above, much of the motivation for developing ESM to focus on these underlying issues is because of the substantial and accruing evidence that a large subset of sufferers of bulimia are particularly impaired in these areas [18], [19], [20], [21], [22], [23], [24], [25], [26]. We hypothesised that this group may benefit preferentially from ESM over CBT. Disappointingly our moderator analysis did not show a differential treatment effect depending on level of socio-emotional impairment at baseline, however the study was probably not adequately powered for this. Moreover, we were unable to collect data on these socio-emotional outcomes at end of treatment and follow-up and future studies of ESM should assess these.

This study had a number of limitations. Our rate of drop-out was high, and this limits the strength of the conclusions we are able to draw. The phenomenon of high drop out is common in bulimia treatment research with rates higher than in other fields [49]. It may also be affected by our inner-city London participant population. Agras et al. [10] compared two treatments for bulimia in Stanford and New York City, and found significantly lower retention rates in NYC. However, the level of drop-out did mean that the study lacked the intended power to enable us to confirm our hypotheses. It will be important in future studies to address this issue to attempt to minimise the level of drop-out.

One particular strength of the study was its ecological validity. It was based in a busy Outpatient clinic in a deprived inner-city area in South London, with all the attendant practical issues associated with such a setting, including stretched clinicians and administrators, and limited practical resources. Also, patients did not receive a monetary compensation and their access to treatment was not dependent on participation in this study. It is encouraging that positive results may be obtained in such a realistic environment.

## Supporting Information

Protocol S1 (DOC)

Checklist S1 (DOC)
