# Supplementary material for: Emotional and Social Mind Training: A Randomised Controlled Trial of a New Group-Based Treatment for Bulimia Nervosa
Source: PLoS One. 2012 Oct 31;7(10):e46047. doi: 10.1371/journal.pone.0046047 (PMC3485274; doi:10.1371/journal.pone.0046047)
Supplement: Protocol S1 — (DOC) [file pone.0046047.s001.doc]

**A Preliminary Randomised Controlled Trial of the Efficacy and Acceptability of a New Emotional and Social Mind Group Training Program vs. Standard Cognitive Behavioural Group Therapy for Bulimia Nervosa.**

**Applicants:** Dr Anna Lavender, Dr Helen Startup, Professor Ulrike Schmidt.

**AIMS OF THE RESEARCH**

- To test the efficacy and acceptability of a new emotional and social mind group training programme for bulimia nervosa (BN) versus a standard cognitive behavioural group therapy programme.
- To explore processes and predictors of outcome in these treatments.
- Specifically, to explore the value of targeting directly difficulties in processing and managing emotion, particularly shame and difficulties in social reasoning, on bulimic symptomatology and self-esteem.

**BACKGROUND**

**The Problem**

Bulimia nervosa (BN) is an eating disorder consisting of recurrent episodes of binge eating followed by compensatory behaviours such as fasting, excessive exercise, or purging, in the context of a general negative evaluation of the self with a specific focus on weight and shape and intolerance of affect. Four to seven percent of young females suffer from full or partial bulimia nervosa (BN) (Hoek *and* van Hoeken, 2003). Typically, BN develops in adolescence and is associated with numerous physical complications and with psychological difficulties, notably anxiety, depression and low self-esteem (see Cooper, 2003, for an overview). Shame is one of the more complex emotions associated with this disorder which can act as a barrier to treatment seeking and engagement (Goss and Gilbert, 2002). Quality of life is poor for sufferers (de la Rie *et al.*, 2005) and the burden on families is high (Perkins *et al*., 2004; Winn *et al*., 2004; 2007). Women with BN who have children may find it hard to feed them and interact with them appropriately (Stein *et al*. 2001**).** Thus BN has a significant cost to sufferers, their families and their offspring.

**Treatment of BN: The Status Quo.**

Over 80 randomised controlled trials have been conducted into the psychological and pharmacological treatment of BN. This research evidence has been summarised in several high quality systematic reviews (National Institute of Health and Clinical Excellence, 2004; Hay *et al.,* 2004). The National Institute of Health and Clinical Excellence (NICE) guidelines (2004) recommend a specific form of cognitive behavioural therapy (CBT-BN) designed by Fairburn and colleagues in Oxford (Fairburn, 1981; Fairburn *et al.*, 1993) to address weight and shape concerns as the first line treatment for this disorder. The recommended treatment dose is 16 to 20 sessions. Individual and group formats of this treatment have been tested, with comparable efficacy (Chen *et al*., 2003). However, with this treatment only approximately 30 to 40 % of people are symptom free at the end of treatment (e.g. Agras *et al*., 2000). The Oxford group have recently updated their CBT treatment to address a broader range of putative maintaining factors (Fairburn *et al*., 2003). However a large RCT (n=154 patients) comparing the more focused (old) version of CBT-BN with the broader (new) version failed to show any difference in outcome (Fairburn, 2007). Thus, there remains an urgent need to develop better treatments for this condition.

**Exploring New Targets for Treatment of BN**

One possibility is that treatment trials have over-focused on targeting the overt symptoms of BN (bingeing and compensatory behaviours) at the expense of the broader intra- and interpersonal attitudes, affects and behaviours typical of the disorder. These factors may be crucial in influencing treatment outcome and/or leaving individuals prone to relapse (e.g. Agras *et al.*, 2000; Openshaw *et al.*, 2004). In a recent systematic review which explored factors predicting poor outcome in eating disorders, two key factors emerged for BN: poor social/interpersonal functioning and negative self-evaluation (Jacobi, 2007). Below we review what is known about these two areas in relation to BN and how this might translate into a new approach to treatment.

*Social/interpersonal functioning and social cognition in BN:*

There is substantial evidence to suggest that women with BN experience difficulties in the social domain during childhood (Troop and Bifulco, 2002; Fairburn *et al*., 1997). Typically they are shy, compare themselves negatively with others and have few friends. Severe life events and chronic difficulties in the social/interpersonal domain trigger the onset of BN in the majority of cases (Schmidt *et al*., 1997; Welch *et al*., 1997).

Women with established BN compare themselves unfavourably to other women and perceive themselves to have a lower social rank than others (Troop *et al*., 2003; Morrison *et al*., 2003). Moreover, they have a more limited social network with fewer supportive relationships (Tiller *et al*., 1997). Pre-existing social difficulties may be worsened by the effects of bulimic symptoms, such as semi-starvation, bingeing or compensatory behaviours (Schmidt *et al*., 1995).

Despite a wealth of literature demonstrating impairments in the social domain in BN, very little work has been done to date on social cognition in BN. Social cognition has been defined as *“the mental operations underlying social interactions, which include the human ability to perceive the intentions and dispositions of others”* (Brothers, 1990). Aspects of social cognition include emotion and social perception, theory of mind and attributional bias. There is some evidence that theory of mind, i.e. the ability to represent the mental states of others in terms of their intentions, desires and beliefs is significantly impaired in patients with BN (Bydlowski *et al*., 2005).

In the treatment literature, Leung *et al*. (2000) found that amongst women treated for BN with group based CBT, it was those who held non-eating disorder-related pathological core beliefs at the outset of treatment who ended up making fewer treatment gains. These included difficulties in the realm of interpersonal functioning and low self-esteem. Thus, there is growing evidence that social functioning and social cognition are impaired in BN and may provide fruitful targets for treatment.

*Negative self-evaluation, shame and social threat*

A consistent finding within the literature concerns the emphasis on affect as a key maintaining factor for BN (Waller *et al.,* 2004). Waller *et al*., (2004) suggest that while anorexia nervosa is characterised by primary avoidance (preventing the experience of distressing cognitions and emotions), BN is characterised by secondary avoidance (removing or blocking the experience of distressing emotions and cognitions). One way of understanding the link between emotion and negative self-evaluation in BN may be through the concept of shame-based self-criticism (Gilbert and Proctor, 2006). In a shame-eliciting situation a person appraises some important aspect of themselves or their behaviour as potentially giving rise to negative appraisal by others and consequently, to a risk of social rejection. Gilbert (2005) hypothesizes that in an attempt to manage this threat of rejection, individuals in a state of shame adopt a mental self-management style characterized by self-criticism, persecution and hostility with the functional intention of prompting behaviour that will reduce rejection threats. Eating disordered individuals have been found to have high levels of both internal shame (self devaluation and self-criticism) and external shame (negative thoughts and feelings about how one exists in the mind of others) (Goss and Gilbert, 2002; Waller *et al*., 1996).

**A New Emotion and Social Mind Training Module**

Taken together the above evidence suggests that negative self-evaluation, poor interpersonal skills, difficulties in understanding the minds of others (the ‘social mind’) and difficulties managing and tolerating emotions, particularly those linked with shame, may be key factors in the maintenance of bulmic symptoms. Based on these ideas we have developed a new emotional and social mind group training program for individuals with BN. We are already using this training module with patients with anorexia nervosa on an individual basis. Preliminary research confirms its utility and acceptability. It is hypothesised that a treatment targeting these factors will lead to positive changes in self-evaluation and interpersonal functioning, and a reduction in bulimic symptoms. Because we know that shame and interpersonal difficulties are central to the pathology of these individuals it is also believed that a group format may provide powerful opportunities to normalise experiences, learn from others and explore the ‘minds’ of others within a safe environment.

**MATERIAL AND METHODS**

**Main hypothesis:**

A 17-session group based intervention (Emotional and social mind training: ESM) targeting the key maintenance variables for bulimia nervosa outlined above will be superior to a 17-session group-based standard CBT programme for this group, in terms of achieving symptomatic improvement in bulimic symptoms and mood.

**Subsidiary hypotheses:**

1. Drop out will be lower in the ESM than the CBT group.
2. Acceptability of treatment will be superior in the ESM group, compared with the CBT programme.
3. ESM will be superior to the CBT programme in terms of the following variables, which will mediate or moderate symptom change:
   1. Reducing intolerance of emotional distress (moderator)
   2. Decreasing negative self-evaluation (mediator)
   3. Increasing adaptive emotional expression and processing (mediator)
   4. Increasing ability to regulate negative mood (mediator)
   5. Reducing self-criticism (mediator)
   6. Reducing negative beliefs about emotions (mediator)
   7. Reducing rumination (mediator)
   8. Reducing socially submissive behaviour (mediator)
   9. Improving social cognition (mediator)

**Design of treatment trial:**

This is a two-arm randomised controlled trial which will evaluate the efficacy of ESM compared to group CBT for adults with BN consecutively referred to the South London and Maudsley NHS Foundation Trust Eating Disorders Service. We will also study processes of and predictors of outcome. Groups will be run by therapists trained in both therapies, and therapists involved will run both groups. Patients in both groups will meet with the research worker to complete the questionnaires and experimental tasks at baseline, at four months, after completing the active treatment, and at six months, after a follow-up period.

**Interventions:**

**Group 1 – Emotional and social mind training (ESM):** Patients in this group will receive a 17-session treatment (4 individual session, 12 group sessions, 1 follow-up session) focusing on the hypothesised maintaining factors outlined above. The individual sessions will be used to develop an individual case formulation. The 12 group sessions will address: identification and understanding of inter- and intrapersonal emotions; the social context of emotions; managing intense and overwhelming emotions; managing shame through compassionate mind training. The follow-up session will be a ‘booster’ for the group. Sessions will take place on a weekly basis. Individual sessions will be 60 and group sessions 90 minutes in length. Group sessions will include eight patients and be facilitated by two therapists. A patient manual for this treatment exists, currently in use within our RCT of individual treatment for patients with anorexia, and can easily be adapted for the group programme.

**Group 2 – Group CBT programme:** Patients in this group will receive a 17-session group-based CBT treatment (4 individual session, 12 group sessions, 1 follow-up session) As with the ESM group, the individual sessions will be used to develop an individual case formulation. The intervention will be based on the group CBT treatment for BN developed by Chen *et al* (2003), adapted from Fairburn *et al* (1993). We chose this programme as the comparison treatment as we wished to match the group format of our ESM intervention whilst comparing it to CBT, the treatment for BN with the strongest evidence base. The timescales and group makeup will be identical to those of the ESM group.

**Outcome measures:**

Research assessments will take place at baseline, 4 months (end of treatment) and 6 months (follow-up).

**Primary outcomes**: Severity of core bulimic symptoms (bingeing, purging, etc…) will be assessed using the Eating Disorders Examination (Fairburn and Cooper, 1993).

**Secondary outcomes:**

Depression, Anxiety and Stress Scale (DASS-21: Lovibond SH and Lovibond PF, 1995); Levels of Self-Criticism Scale (LOSC: Thompson *and* Zuroff, 2004). Distress Tolerance Scale (DTS: Corstophine *et al*., 2007); Beliefs About Emotions Scale (Rimes and Chalder, in preparation); Forms of the Self-Criticizing/Attacking and self-Reassuring Scale (FSCRS: Gilbert *et al*. 2004); Functions of the Self-Criticizing/Attacking Scale (FSCS: Gilbert *et al*. 2004); Submissive Behaviour Scale (SBS: Allan *and* Gilbert, 1997); Rumination Questionnaire (Watkins *and* Barnard, in preparation). Participant satisfaction will be assessed using visual analogue scales. Social cognition tasks: Reading the Mind from the Eyes Test (Baron-Cohen and Hammer (1997); Reading the Mind in Films Task (Golan *et al*., 2006); Interpersonal Perception Task-15 (IPT-15: Costanzo *and* Archer, 1993).

**Randomisation, Blinding and Protection against Bias:**

At the point of referral patients’ suitability for participation will be checked by a clinician. If the patient is suitable and consents, she/he will be introduced to the research assessor who will complete the research assessment. The patient will then be randomised by a computerised system. Stratified randomisation will be used, stratifying for diagnosis (BN or EDNOS) to assign patients to one of the two trial arms. Patients will be told about the outcome of randomisation by the clinician. Throughout the trial every effort will be made to ensure that the assessor remains blind to the treatment condition. All therapeutic sessions will be taped and a random selection of these listened to by the research team to check for adherence to therapeutic model and quality of intervention.

**Inclusion/exclusion criteria:**

Inclusion: patients referred to the SLAM Eating Disorder Service who fully (BN) or partially (EDNOS) fulfil DSM-IV criteria for bulimia nervosa.

Exclusion: insufficient knowledge of English or literacy levels to allow understanding of the intervention materials, active suicidality, severe substance dependence, diabetes or pregnancy. Informed written consent will be sought from patients at initial assessment.

**Proposed sample size:** Because ESM is a novel intervention, power was calculated on the basis of Chen *et al* (2003). A sample size of 30 in each arm of the study will have 80% power to detect a difference in means of -0.850 on the EDEQ Global score assuming that the common standard deviation is 1.15 using a two group t-test with a 0.05 two-sided significance level. Assuming a drop-out rate of 20% a sample size of 76 patients will be needed.

**STATISTICAL TREATMENT OF RESULTS**

All outcomes will be analysed on an intention to treat basis including all randomised patients. Analyses will also be performed on a ‘per protocol’ basis (including only those who complete treatment) as a sensitivity analyses. Efficacy: We will employ multi-level modelling analyses for missing data using maximum likelihood methods (Everitt, 1998) to compare bulimic symptoms within and between groups. We will control for potential confounding variables (e.g. symptom levels at baseline), by using them as covariates in the analysis. Drop out rates will be compared across the two treatment groups using Pearson’s chi-square test. Predictors and Processes: Regression methods will be used to explore the relationship between potential predictor variables as described above in the hypotheses section and outcomes separately in the two groups. Treatment acceptability: patient satisfaction will be compared across the two treatment groups using t-tests or Mann-Whitney U tests, depending on the distribution.

**JUSTIFICATION OF BUDGET**

One research worker (grade 1B) will be required for eighteen months for the day-to-day running of the study. The research worker will recruit patients from all four sites of the Maudsley EDU and conduct baseline assessments, end of treatment assessments and follow-up assessments. Total salary cost: approximately £52,564. Additional costs include: office costs, £2000; equipment, £3000. Total cost of project: £57,564.

References

Adolphs, R. (1999). Social cognition and the human brain. *Trends in Cognitive Science*, 3, 469-479.

Agras, W., Stewart, W. T., Fairburn, C. G, Wilson, G. T. & Kraemer, H. C. (2000). A multicenter comparison of cognitive-behavioral therapy and interpersonal psychotherapy for bulimia nervosa. *Archives of General Psychiatry*, 57(5), 459-466.

Allan, S., & Gilbert, P. (1997). Submissive behaviour and psychopathology. *British Journal of Clinical Psychology*, 36, 467-488.

American Psychiatric Association (1994). *Diagnostic and Statistical Manual of Mental Disorders (4th ed.).* Washington: American Psychiatric Association.

Bailer, U., de Zwaan, M., Leisch, F., Strnad, A., Lennkh-Wolfsberg, C., El-Giamal, N., Hornik, K. & Kasper, S. (2004). Guided self-

help versus cognitive-behavioral group therapy in the treatment of bulimia nervosa. *International Journal of Eating Disorders*, 35, 522-537.

Baron-Cohen & Hammer (1997). Parents of children with Aspergers syndrome: what is the cognitive phenotype? *Journal of Cognitive Neuroscience*, 9, 548-554.

Brothers, L. (1990). The social brain: a project for integrating primate behaviour and neurophysiology in a new domain. *Concepts in Neuroscience*, 1, 27-51.

Bydlowski, S, Corcos, M, Jeammet, P., Paterniti, S., Berthoz, S., Laurier, C. Chambry, J., Consoli, S. M. (2005) Emotion-Processing Deficits in Eating Disorders. *International Journal of Eating Disorders. Vol 37(4)*, 321-329.

Catanzaro, S.J., & Mearns, J. (1990). Measuring generalized expectancies for negative mood regulation: Initial scale development and implications. *Journal of Personality Assessment*, 54, 546–563.

Chen, E., Touyz, S., Beumont, P., Fairburn, C., Griffiths, R., Butow, P., Russsell, J., Schotte, D., Gertler, R., Basten, C (2003). Comparison of group and individual cognitive-behavioural therapy for patients with bulimia nervosa. International Journal of Eating Disorders, 33 (3), 241-254.

Cooper, M. J. (2003). *Bulimia nervosa: A cognitive perspective*. Oxford: Oxford University Press.

Corstophine, E, Mountford, B, Tomlinson, S, Waller, G & Meyer, C (2007). Distress tolerance in the eating disorders. *Eating Behaviours,* 8(1), 91-97.

Costanzo, M & Archer, D (1993). *The Interpersonal Perception Task -15 (IPT-15). A guide for researchers and teachers.* University of California Extension, Berkeley, California.

de la Rie, S. M, Noordenbos, G & van Furth, E. F. (2005). Quality of life and eating disorders. *Quality of Life Research: An International Journal of Quality of Life Aspects of Treatment, Care & Rehabilitation*. 14(6), 1511-1522.

Everitt, B.S. (1998). Analysis of longitudinal data. Beyond Manova. *British Journal of Psychiatry, 172*, 7-10.

**Fairburn, C (1981). A cognitive behavioural approach to the management of bulimia. *Psychological Medicine*, 11, 707-711.**

Fairburn, C.G. & Cooper, Z. (1993). The eating disorders examination. In C. Fairburn & G. Wilson (Eds.), *Binge eating: Nature, assessment, and treatment*. New York: Guilford.

Fairburn, C. G., Cooper, Z., & Shafran, R. (2003). Cognitive behaviour therapy for eating disorders: A transdiagnostic theory and treatment. *Behaviour Research and Therapy*, 41, 509–528.

**Fairburn, C., Marcus, M. & Wilson, G. (1993). Cognitive-behavioural therapy for binge eating and bulimia nervosa: A comprehensive treatment manual. In C. Fairburn & G. Wilson (Eds.) *Binge eating: nature, assessment and treatment.* New York: Guildford.**

Fairburn, C. G., Norman, P. A., Welch, S. L., O’Connor, M. E., Doll, H. A. & Peveler, R. C. (1995). A prospective study of outcome in bulimia nervosa and the long term effects of three psychological treatments. *Archives of General Psychiatry*, 52, 304–312.

Gilbert, P. (Ed) (2005). *Compassion: Conceptualisations, Research and Use in Psychotherapy*. Routledge, Hove, UK.

Gilbert, P. & Andrews, B. (1998). (Eds) *Shame: Interpersonal Behaviour, Psychopathology and Culture*. New York: Oxford University Press.

Gilbert, P., Clarke, M., Kempel, S., Miles, J., & Irons, C. (2004). Criticizing and reassuring oneself: An exploration of forms style and reasons in female students. *British Journal of Clinical Psychology*, 43, 31-50.

Gilbert, P. & Procter, S. (2006). Compassionate mind training for people with high shame and self-criticism: Overview and pilot study of a group therapy approach. *Clinical Psychology and Psychotherapy*, 13, 353-379.

Golan, Ofer; Baron-Cohen, Simon; Hill, Jacqueline. (2006). The Cambridge Mindreading (CAM) Face-Voice Battery: Testing Complex Emotion Recognition in Adults with and without Asperger Syndrome. *Journal of Autism and Developmental Disorders. Vol 36(2)*, 169-183.

Goss, K. & Gilbert, P. (2002). Eating disorders, shame and pride. A cognitive-behavioural functional analysis. In, P. Gilbert & J. Miles (Eds.). *Body Shame*. Brunner-Routledge, Hove, UK.

Hay P.J., Bacaltchuk J. & Stefano S. (2004). Psychotherapy for bulimia nervosa and binging. *Cochrane Database of Systematic Reviews,* Issue 3.

Heimpel, S.A., Wood, J, A, Marshall, M.A., & Brown, J.D. (2002). Do people with low self-esteem really want to feel better? Self-esteem differences in motivation to repair negative moods. *Journal of Personality and Social Psychology*, 82, 128-147.

Hoek, H. & van Hoeken, D. (2003). Review of the prevalence and incidence of eating disorders. *International Journal of Eating Disorders. Vol 34(4),* 383-396.

Jacobi, C. (2007). Risk factors for eating disorders. In Wonderlich, S. & Mitchell, J. (Eds.), *Academy of Eating Disorders Annual Review of Eating Disorders.*

Katzman, M.A., Bara-Carril, N., Rabe-Hesketh, S. Schmidt, U.,deSilva, P., Troop, N.,  Treasure, J. (2007). A randomized controlled two-stage trial in the treatment of bulimia nervosa, comparing CBT versus motivational enhancement in phase 1 followed by group versus individual CBT in phase 2. submitted for publication.

Keys A., Brozek J., Henschels A., Mickelsen O. & Taylor H. (1950). *The Biology of Human Starvation*. University of Minnesota Press, Minneapolis.

Lehman, A.K. & Rodin, J. (1989). Styles of self-nurturance and disordered eating. *Journal of Consulting and Clinical Psychology*, 57, 117-122.

Leung, N., Waller, G. & Thomas, G. (2000). Outcome of group cognitive-behaviour therapy for bulimia nervosa: the role of core beliefs. *Behaviour Research and Therapy*, 38, 145-156.

Meyer, C. & Waller, G. (1999). The impact of emotion upon eating behaviour. The role of subliminal visual processing of threat cues. *International Journal of Eating Disorders*, 25, 319- 326.

Morrison *et al*., (2003). Social comparison in eating disorders. *Journal of Nervous and Mental Disease,* 191 (8), 553-5.

National Institute for Clinical Excellence. (2004). Eating Disorders: Core interventions in the treatment and management of anorexia nervosa, bulimia nervosa and related eating disorders. *Clinical Guideline 9*. London: National Institute for Clinical Excellence.

Nevonen, Lauri; Broberg, Anders G. (2006). A comparison of sequenced individual and group psychotherapy for patients with bulimia nervosa. *International Journal of Eating Disorders. Vol. 39(2)*, 117-127.

Openshaw, C., Waller, G., Sperlinger, D. (2004). Group cognitive-behaviour therapy for bulimia nervosa: Statistical versus clinical significance of changes in symptoms across treatment. *International Journal of Eating Disorders, 36*, 363-375.

Patton, C.J. (1992). Fear of abandonment and binge eating. A subliminal psychodynamic activation investigation. *Journal of Nervous and Mental Disease, 180*, 484-490.

Perkins, S., Winn, S., Murray, J., Murphy, R., Schmidt, U. (2004). A Qualitative Study of the Experience of Caring for a Person with Bulimia Nervosa. Part 1: The Emotional Impact of Caring. *International Journal of Eating Disorders. Vol. 36(3),* 256-268.

Rimes & Chalder. (In preparation). Beliefs about emotions questionnaire.

Roberts, M.E., Lopez, C., Tchanturia K. and Treasure, J. (2007). Sudden treatment gains in a new neurocognitive feedback module for anorexia nervosa.  Submitted.

Russell, T.A., Schmidt U., Doherty E., Young V., Tchanturia K. (2007).  Aspects of Social Cognition  in Anorexia Nervosa: A Study of ‘Hot’ and ‘Cold’ Theory of Mind Tasks. In preparation.

Schmidt, U.H., Evans, K., Tiller, J., & Treasure, J. (1995). Puberty, sexual milestones and abuse: How are they related in eating disorder patients? *Psychological Medicine, 25*, 413–417.

Schmidt, U.H., Humfress, H. & Treasure, J.L. (1997). The role of general family environment and sexual and physical abuse in the origins of eating disorders. *European Eating Disorders Review, 5,* 184-207.

Schmidt, U., Tiller, J., & Treasure, J. (1993). Psychosocial factors in the origins of bulimia nervosa. *International Review of Psychiatry, 5*, 51–60.

Schmidt, U; Landau, S; Pombo-Carril, M; Bara-Carril, N; Reid, Y; Murray, K; Treasure, J; Katzman, M. (2006). Does personalized feedback improve the outcome of cognitive-behavioural guided self-care in bulimia nervosa? A preliminary randomized controlled trial. *British Journal of Clinical Psychology. Vol 45(1)*, 111-121.

Stanton, A.L., Danoff-Burg, S., Cameron, C., Bishop, M., Collins, C.A., Kirk, S.B., Sworowski, L.A., & Twillman, R. (2000). Emotionally expressive coping predicts psychological and physical adjustment to breast cancer. *Journal of Consulting and Clinical Psychology, 68*, 875 - 882.

Stein, A., Woolley, H., Murray, L., Cooper, P. Cooper, S., Noble, F., Affonso, N. Fairburn, Christopher, G. (2001) Influence of psychiatric disorder on the controlling behaviour of mothers with 1-year-old infants: A study of women with maternal eating disorder, postnatal depression and a healthy comparison group. *British Journal of Psychiatry. Vol 179* 157-162.

Stefano, S., Bacaltchuk, J., Blay, S., Hay, P. (2006). Self-help treatments for disorders of recurrent binge eating: a systematic review. *Acta Psychiatrica Scandinavica 113 (*6).

Tagney, J.P., Wagner, P. & Gramzow, R. (1989). Proneness to shame, proneness to guilt, and psychopathology. *Journal of Abnormal*

*Psychology, 101*, 469-478

Tchanturia, K; Happe, F., Godley, J., Treasure, J., Bara-Carril, N; Schmidt, U. (2004). 'Theory of Mind' in Anorexia Nervosa.

*European Eating Disorders Review. Vol. 12(6)*, 361-366.

Thompson, R & Zuroff, D. (2004). The levels of self-criticism scale: comparative self-criticism and internal self-criticism.

*Personality and Individual Differences, 36 (2),* 419-430.

Tiller *et al*, (1997). Social Support in Patients with Anorexia Nervosa and Bulimia Nervosa. *International Journal of Eating*

*Disorders, Vol. 21(1)*, 31–38

Treasure, J.L. & Owen, J.B. (1997). Intriguing links between animal behaviour and anorexia nervosa. *International Journal of Eating*

*Disorders, 21,* 307–311

Treasure, J; Tchanturia, K; Schmidt, U. (2005). Developing a model of the treatment for eating disorder: Using neuroscience research

to examine the how rather than the what of change. *Counselling & Psychotherapy Research. Vol. 5(3)*, 191-202.

Troop, N.A. & Bifulco, A. (2002). Childhood social arena and cognitive sets in eating disorders. Responses to life events and

difficulties. *British Journal of Medical Psychology, 70*, 373-385.

Troop, N.A. *et. al*., (2003). Social comparison and submissive behaviour in eating disorder patients. *Psychology and Psychotherapy:*

*Theory, Research and Practice, 76*, 237–249

Tyrer, P., Nur, U., Crawford, M., Karlsen, S., McLean, C., Rao, B. (2005). The Social Functioning Questionnaire: a rapid and robust

measure of perceived functioning*. International Journal of Social Psychiatry, 51*:265-75.

Waller, G, Watkins, H Shuck, V and McManus, F (1996). Bulimic psychopathology and attentional biases to ego threats among non-

eating-disordered women. *International Journal of Eating Disorders, 20(2)* 169-176.

Waller, G., Kennerley, H., & Ohanian, V. (2004). Schema-focused cognitive behaviour therapy with the eating disorders. In P. du Toit (Ed.), *Cognitive schemas and core beliefs in psychological problems: A scientist practitioner guide*. Washington: American Psychiatric Association.

Winn, S., Perkins, S., Murray, J., Murphy, R., Schmidt, U. (2004). A qualitative study of the experience of caring for a person with bulimia nervosa. Part 2: Carers' needs and experiences of services and other support. *International Journal of Eating Disorders. Vol. 36(3),* 269-279.

Winn, S., Perkins, S., Walwyn, R., Schmidt, U., Eisler, I., Treasure, J., Berelowitz, M., Dodge, L., Frost, S., Jenkins, M., Johnson-Sabine, E., Keville, S., Murphy, R., Robinson, P., Yi, I. (2007). Predictors of mental health problems and negative caregiving experiences in carers of adolescents with bulimia nervosa. *International Journal of Eating Disorders. Vol. 40(2),* 171-178.

Zigmond, AS., & Snaith, RP. (1983). The hospital anxiety and depression scale. *Acta. Psychiatr. Scand., 67,* 361-70.
